# The pandemic paradox: domestic violence and happiness of women

**DOI:** 10.7717/peerj.10472

**Published:** 2020-11-24

**Authors:** Wajiha Haq, Syed Hassan Raza, Tahir Mahmood

**Affiliations:** 1 Department of Economics, School of Social Sciences and Humanities, National University of Sciences and Technology, Islamabad, Pakistan; 2 School of Economics, Quaid-i-Azam University, Islamabad, Pakistan; 3 Department of Technology, School of Science and Technology, The Open University of Hong Kong, Kowloon, Hong Kong

**Keywords:** Married, Women, COVID-19, Violence, Lockdown, Happiness

## Abstract

**Background:**

Across the globe, lockdowns have been enforced as a pandemic response to COVID-19. Such lockdown coupled with school closures and stay-at-home orders made women more vulnerable in terms of higher responsibility and spending more time with an abusive partner, if any.

**Methods:**

This study investigates the situation of women during COVID-19 induced lockdown by focusing on their happiness and inquiring about the incidence of violence. Using the zero-inflated negative binomial model, our findings ascertained that family settings, type of relationship with a spouse, and age significantly affects the positive count of violence during the lockdown. We further estimated the determinants of happiness and found that years of schooling, the role of women in household decision making, and feeling empowered is affecting their happiness.

**Results:**

Women having higher education have more odds of zero violence. Unemployed women and women who are not working have higher odds of zero violence as compared to women who are working. During this lockdown after the COVID-19 pandemic, women living in urban areas, having higher education, having an adequate household income to meet the expenditures, having lesser anxiety, not facing violence, feeling empowered when their husband is around, and have higher decision-making power are happier.

**Discussion and conclusion:**

The study is important in the context of happiness and violence inflicted on women during the lockdown and provides the basis to improve the pandemic response policy. The inclusion of women’s safety and happiness in pandemic response policy is important to ensure the well-being of women and to devise better health and economic policy. Our estimates suggest higher education results in less incidence of violence which could be argued as desirable outcomes for building healthy, productive, and happy communities. In addition to this, as pandemic induced lock-down is likely to result in higher unemployment across the globe including Pakistan, therefore, in light of our estimates pertaining to the role of unemployment in the incidence of violence, policymakers should deploy more resources to enhance income and to combat the rising unemployment. As a counter-intuitive outcome of these policy interventions, incidence of violence will be dampened, educational attainment and women empowerment will be increased which will certainly increase happiness.

## Introduction

The world is facing the pandemic of COVID-19, which is the greatest global health crisis of our time. The pandemic is affecting people across all the age groups, gender, or ethnicities which exacerbates the psychological, social, and economic repercussions. The pandemic induced lockdown has exposed the underlying vulnerabilities of women as they are forced to stay at home, work from home, and implement social distancing. This situation reduces women’s autonomy especially in patriarchal homes which is also evident from the increase in incidents of domestic violence incidents across the globe (Peterman et al., 2020). Therefore, it is noteworthy to document whether the COVID-19 is a double pandemic for women or not in a context. If a woman goes out, she is vulnerable, but the question remains for her safety if she stays at home. Besides, it is also imperative to capture the extent of her happiness while staying at home. Such an assessment is pivotal to devise better and effective economic, health, and social welfare policy.

The COVID-19 made women vulnerable to all sorts of gender-based violence. The rates of violence against women increased across the globe during the pandemic (Pfitzner, Fitz-Gibbon & True, 2020; Ravindran & Shah, 2020). The COVID-19 pandemic and accompanying increase in violence against women has been termed as a shadow pandemic by United Nations Women (2020). The lockdown situation during the pandemic even worsened the situation for the women (Sánchez et al., 2020). In Pakistan, the reports of violence against women increased exponentially (United Nations Office on Drugs & Crime, 2020).

Of note, the World Bank (2020) also called to investigate the impact of COVID-19 induced lockdown on the women given the issues related to empowerment, happiness, and violence against women.

In Pakistan, as of May 30th, 22.48% of women have been infected by the COVID-19 with the highest prevalence rate among the age group of 20–29 years (OCHA Pakistan, 2020). Apparently, women seem less infected by the pandemic as higher mortality is observed among men due to more prevalence of smoking and gender-based immunological differences (Wenham, Smith & Morgan, 2020). Governments across the globe have implemented lockdowns, enforced people to stay at home and work from home, and further announced the complete closure of educational and non-educational institutes. Due to culturally imposed roles of women and patriarchal norms in many countries, the wellbeing of women is adversely affected as they are at the forefront as unpaid caregivers or front line workers even during the pandemic (Gibbs et al., 2020). The domain of adverse effects on women is broad, which primarily includes the risk of violence, ill-being, and adverse psychological health. Furthermore, it has been widely argued that the effects of economic slow-down bring more severe consequences to women compared to men in the context of job security and wage differences (De Paz et al., 2020). Even the wellbeing of unpaid caregivers (housewives) is influenced through the instability in the economic outcomes of households along with various other sociological and psychological outcomes. On the face of the outcomes, the vulnerability of women during the COVID-19 induced lockdown becomes an even more important phenomenon to capture.

As of May 30, 2020, the majority of the countries including Pakistan are still under some form of partial or complete lockdown. Thus, it could be argued that the economic turmoil induced by lockdown coupled with restrictions on mobility and social isolation may expose women to domestic violence. In particular to Pakistan, on an average basis, one in four women suffers from emotional, physical, or sexual violence (National Institute of Population Studies-NIPS/Pakistan and ICF, 2019). This implies that at least eight million women grapple with various forms of violence every year and Pakistan is already argued as the sixth most dangerous country for the women (Thomson Reuters Foundation, 2018). As a social norm, the majority of the women in Pakistan are not particularly active in the labor market; thus, staying home and performing daily household chores are their routine but a significant number goes to work for daily livelihood. Under such circumstances, whether working or not, when the government has induced lock-down staying at home with an abusive partner during the COVID-19 induced lockdown makes them more vulnerable, and this certainly affects their wellbeing as well. Safdar & Yasmin Alvi (2020) argued, *“the chances of the vulnerability of the negotiated identity and social space of middle-class educated Muslim women in urban Pakistan get heightened in situations like COVID-19 mainly due to the religiously inspired dominant patriarchal social behaviors and the state’s inability to practically empower its women during normal conditions”*.

Those women who are already marginalized with limited economic resources, overburdened household responsibilities, and taking care of children are more susceptible to violence while spending more time with an abusive partner during the COVID-19 induced lockdown with dismal support (United Nations, 2020). This sort of lockdown situation dampens their ability to look for other alternatives and results in accepting the violence with lower happiness.

It is noteworthy that under varied circumstances, women not only accept the violence but considers it normal (García-Moreno et al., 2005). Women mostly work in the informal sector and are excluded from formal social insurance and protection especially in the developing world (De Paz et al., 2020). Besides, the responsibility of taking care of children and older people also falls on her shoulders. United Nations (2020) in its policy brief about the impact of COVID-19 on women mention that on average, women spend 4.1 h/day on unpaid work including domestic chores and care provision compared to 1.7 h/day by men. As far as unpaid contributions from women are concerned, they constitute 2.35% of the total GDP. Across all the age groups, women take care of the elderly. Therefore, they are considered as one of the most important pillars of healthcare. Past outbreaks have shown that promoting gender roles increase the efficacy of health interventions (Wenham, Smith & Morgan, 2020). At the intersection of above-mentioned ideas and debates, it is pivotal to capture the violence and happiness of the women during the COVID-19 induced lockdown in Pakistan so that an effective pandemic response including women, ensuring their safety and wellbeing, can be devised and they can play an active and effective role in the fight against COVID-19.

## Materials and Methods

### Questionnaire design

To investigate the situation of women during the lockdown in this pandemic, a survey is designed based on literature (De Paz et al., 2020; Ewerling et al., 2017; UNHCR-IDC, 2016; World Bank, 2020; Zhang et al., 2020) for married women. Our survey has gone through various checks, including a detailed discussion on the design of the survey and ethical standards by the experts. The National University of Science and Technology, Pakistan granted ethical approval for the study (Ethical approval Letter Ref: 0801/02/fac-offr/s3h). Participants were informed about the purpose of the questionnaire that they are going to be filled and the anonymity of respondents was assured. In addition to this, we did not ask any personal details such as name, address, contact or national identity number. Filling out the questionnaire and submitting it was their informed consent. Furthermore, we initially launched the pilot survey in the last week of May 2020, and later, after the necessary corrections, we fielded the survey in the first 3 weeks of June 2020. The main intent of the survey remains about documenting the responses of such women who are tech aware to fill up the survey. Although we understand in this situation we will have a majority of the responses from the women who are truly educated to use the technology. Nonetheless, if they are facing a double pandemic including COVID-19, lockdown induced violence, and loss of happiness so our findings remain applicable to those who are not really tech aware and perhaps not too educated as well due to the overall dismal situation of Pakistan in terms of gender parity and violence against women. The overall indicators for violence against women are in jeopardy. According to (United Nations Office on Drugs & Crime, 2020), 90 percent of women in Pakistan have faced some form of domestic violence. Our study, although based on technologically advanced women, also has implications for women who are not technologically advanced as they will certainly be at least in the same situation or worse than this.

We rely on snowball sampling and conducted the online survey because face to face interviews are not possible due to the ongoing lockdown situation, therefore, focusing on such women which are tech aware and able to fill the online survey serves the need. Google forms are used to make the online survey and respondents are shared the link to fill the questionnaire.

The survey covered the demographic, economic, socioeconomic, and psychological aspects of married women. In addition to this, the respondents are asked to provide information on the technological awareness, relationship with husband, self-perceived empowerment, role in household decision making, violence, and happiness during the COVID-19 induced lockdown. Demographic variables of married women include region (urban/rural), age (years), education (years of education), employment status (full time working, part-time working, not working and unemployed), her husband’s age, husband years of education, husband’s employment status, amount of her salary, and the number of children. Women were also asked about the kind of family setting they live in, which includes nuclear family, extended family, and multiple family settings. The nuclear family was operationally defined as the family which includes husband, wife, and children. The extended family was operationally defined as a family having a husband, wife, children, grandchildren, their parents, and any unmarried siblings living together in the same house. Multiple families were operationally defined as the family having husband, wife, children, grandchildren, their parents, parents-in-law, married siblings, and their children living together in the same house.

Women were also asked to rate their relationship with their husbands on a Likert scale ranging between very poor (coded as 1) and very good (coded as 5). Women’s perception regarding their empowerment was also captured before and after the pandemic. They were asked about how much they feel that they were empowered before COVID-19 to which they responded on a seven-point scale ranging between not very empowered to very empowered. The respondents also provided the information whether they feel empowered when their husband is around to which they responded using a Likert scale ranging between strongly disagree to strongly agree. The psychological effect of the pandemic on women was also investigated by asking women about having anxiety after the pandemic. The same five-point Likert scale of the agreement was used to get the responses.

Sufficiency of household income during COVID-19 related lockdown, access to the police for seeking help during the violence, and access to personal protection equipment (hand gloves, face masks, and hand sanitizers) were also asked. During the lockdown, women are in trouble because all the responsibility falls on their shoulders. School closures have also brought extra responsibility of teaching children at home to her. In this context, women were asked that do they take care of children alone, with a husband or husband takes care of them, or she seeks the help of someone else. The same question was rephrased for performing household chores in order to see who is mainly responsible for taking care of children and performing household chores.

Women’s decision making was captured through three dimensions. Their decision making in economic matters, health spending, and social distancing captured through a five-point Likert scale was used to construct the variable. Access to personal protection equipment was measured through three items, namely access to hand gloves, face mask, and hand sanitizers which were responded with Yes/No. The reliability of items to measure the variables was checked through Cronbach Alpha. The Cronbach Alpha of women’s decision making was 0.75, and access to personal protection equipment was 0.78. The The Cronbach alpha of violence is 0.8. Following the reliability criteria Bland & Altman (1997), the items were valid to measure the phenomena as their values lied between 0.70 and 0.90. We can say that the instrument used to measure the phenomenon is valid (Wang et al., 2020) and generalized with a 5% chance of error. Since the main focus of the survey is to capture the dynamics related to COVID-19 induced lockdown in the context of violence, empowerment, and happiness of the women, thus, the measurement of violence and happiness is explained as follows.

#### Women violence

Violence is a worldwide phenomenon, and during this lockdown, World Health Organization along with many other researchers have shown the concern that the prevalence of women violence may increase during this lockdown (Peterman et al., 2020; Safdar & Yasmin Alvi, 2020; United Nations, 2020; World Bank, 2020). Since the phenomena of violence may take various forms such as physical, emotional, and verbal abuse. The definition of violence was derived from the literature (World Health Organization, 2005). Thus, we asked the count (i.e., the number of times) the respondent (married women) faced any violence (physical, emotional, and verbal) during this lockdown. In addition to this, we further asked her (Yes/No) if she experienced a similar type of violence before such a lockdown. We are particularly interested to know what is happening with her during the lockdown but it is important to understand if the similar situation prevailed before the lockdown.

#### The overall happiness of women

COVID-19 has not only affected people with disease directly but also affected their mental health indirectly. Their indirect effects are far more than the direct effects. The pandemic has affected the mental health of people including women because of boredom, frustration, and fear of getting the disease (Brooks et al., 2020). In addition to this, as we discussed earlier that women are at increased risk of violence during the pandemic induced lockdown, thus, it is right to argue that it can definitely effect the subjective happiness of those women. As happiness comes from stronger and healthier relationships with friends, families, and partners in addition to other sources (Diener & Seligman, 2002) whereas poor social relationships have a negative impact on happiness. An empirical study in Nicaragua investigated that women having episodes of abuse are not very happy (Vázquez, Panadero & Rivas, 2015).

The respondents were asked that overall, how much they feel happy about their life before and after the pandemic which we operationally defined as overall subjective happiness. A seven-point scale was used to gauge happiness (Chinni, 2014) which ranges from not very happy (coded as 1) to very happy (coded as 7).

### Data collection

The data was collected through a snowball sampling technique where the questionnaire was floated among few married women, and they were asked to share this questionnaire with other married women they know as well. The mode of data collection was online to ensure safety and to avoid any inconvenience for the respondents during the lockdown. Through online data collection, we also did not breach the standard operating procedures of social distancing advised by the Government of Pakistan. During this situation, the best possible way to collect data is through the online surveying approach(Wang et al., 2020). The online data collection helped us to spread our reach to married women across Pakistan. We collected the data from 412 married women, and after the necessary data cleaning for missing observations, we retained 389 responses. Therefore, with a 3% margin of error and a 95% confidence level, our data is a national representative. Women in our sample belong to 54 distinct districts of Pakistan which prove that the regional variation is significantly covered in our survey.

### Analysis technique

#### The zero-inflated negative binomial model

The data of violence is collected in the form of the number of violence during the lockdown, where most of the women reported no violence (i.e., zero counts). There exist several discrete distributions to model count data such as Poisson, Negative Binomial, zero-inflated Poisson (ZIP), and zero-inflated Negative Binomial (ZINB) distribution. For the modeling, the first step is to know the best-fitted distribution of the dependent variable, and for such purpose, we have fitted all competitive count distributions, and computed their results. Based on the results, an excess number of zeros and over-dispersion, we reached to the conclusion that ZINB is the best-fitted distribution to model the number of violence. Due to the excess number of zeros and over-dispersion, we assessed that the ZINB distribution is the best-fitted model for the violence (see “Results” section). The ZINB distribution comprises two processes, where one is used to model the nonzero counts (i.e., ${y} > 0$) while the other is used to fit the zero counts (i.e., $y = 0$). Hence, the probability mass function of the ZINB can be described as follows:

$$f\left( {y;p,{\rm \lambda },{\rm \tau}} \right) = \left\{ {\matrix{ {\left( {1 - p} \right) + p \times {{\left( {\displaystyle{\rm \tau \over {\rm \tau + {\rm \lambda }}}} \right)}^{\rm \tau} }} & {y = 0} \cr {p \times \displaystyle{{{\Gamma }\left( {y + \rm \tau } \right)} \over {y!{\Gamma }\left( \rm \tau \right)}}{\rm \; }{{\left( {\displaystyle{\rm \tau \over {\rm \tau + {\rm \lambda }}}} \right)}^{\rm \tau} }{{\left( {\displaystyle{{\rm \lambda } \over {\rm \tau + {\rm \lambda }}}} \right)}^y}} & {y = 1,2, \ldots ,{\rm \; }} \cr } } \right.,$$

where ${\rm \lambda }$ is the mean of the underlying Negative Binomial distribution, $\rm \tau$ is the over-dispersion parameter and $p$ is the probability of zero observations. It is to be noted that when $\rm \tau \to 0$, the ZINB model reduced to the ZIP distribution, when $p \to 0$, the ZINB model reduced to the Negative Binomial distribution, and when both $1/\rm \tau$ and ${P_i} \approx 0$ the ZINB distribution also reduced to the ordinary Poisson distribution.

In order to see the factors affecting the violence on women during this lockdown, the ZINB regression is used to model the data. The ZINB regression model is defined as follows:

$$P\left( {{Y_i} = {y_i}{\rm |}{X_i},{Z_i}} \right) = \left\{ {\matrix{ {\left( {1 - {p_i}} \right) + {p_i} \times {{\left( {\displaystyle{\rm \tau \over {\rm \tau + {{\rm \lambda }_i}}}} \right)}^{\rm \tau} }} & {{y_i} = 0} \cr {{p_i} \times \displaystyle{{{\Gamma }\left( {{y_i} + \rm \tau } \right)} \over {{y_i}!{\Gamma }\left( \rm \tau \right)}}{\rm \; }{{\left( {\displaystyle{\rm \tau \over {\rm \tau + {{\rm \lambda }_i}}}} \right)}^{\rm \tau} }{{\left( {\displaystyle{{{{\rm \lambda }_i}} \over {\rm \tau + {{\rm \lambda }_i}}}} \right)}^{{y_i}}}} & {{y_i} = 1,2, \ldots ,{\rm \; }} \cr } } \right.,$$

where ${X_i}$ is the vector of explanatory variables and ${Z_i}$ is the vector of covariate used to define the probability of excess zero ${P_i}$. The conditional mean and variance of the ZINB regression model are $E\left( {{Y_i}{\rm |}{X_i},{Z_i}} \right) = \left( {1 - {P_i}} \right){\lambda _i}$ and $\; Var\left( {{Y_i}{\rm |}{X_i},{Z_i}} \right) = {\lambda _i}\left( {1 - {P_i}} \right)\left( {1 + {P_i}{\lambda _i} + {\lambda _i}/\rm \tau } \right)$. The parameters ${\lambda _i}$ and ${P_i}$ are defined as follows:

$${\lambda _i} = {exp}\left( {X_i^{\prime}\rm \beta } \right);\; {P_i} = \displaystyle{{{exp}\left( {Z_i^{\prime}\rm \gamma } \right)} \over {1 + {exp}\left( {Z_i^{\prime}\rm \gamma } \right)}},$$

where the vectors $X_i^{\prime} = {x_{i,0}},{x_{i,1}}, \ldots ,\; {x_{i,m}}$ contains $m$ covariates and ${\rm \beta }^{\prime} = \left( {{\rm \beta _0},\; {\rm \beta _1},\; \ldots ,\; {\rm \beta _m}} \right)$ is the vector of $m$ unknown parameters. However, the vector $Z_i^{\prime} = {z_{i,0}},{z_{i,1}}, \ldots ,\; {z_{i,m}}$ contains $m$ covariates for defining the probability of excess zero ${P_i}$ and ${\rm \gamma }^{\prime} = \left( {{\rm \gamma _0},\; {\rm \gamma _1},\; \ldots ,{\rm \gamma _m}} \right)$ is the vector of $m$ unknown parameters. The ZINB log-likelihood given the observed data is obtained as follows:

$$\eqalign{l\left( {Y,\rm \beta ,\rm \gamma ,\rm \tau } \right) &= \mathop \sum \limits_{i = 1}^n \ln \left( {1 + {e^{Z_i^{\prime}\rm \gamma }}} \right) - \mathop \sum \limits_{i:{y_i} = 0} \ln \left( {{e^{Z_i^{\prime}\rm \gamma }} + {{\left( {\displaystyle{{{e^{X_i^{\prime}\rm \beta }} + \rm \tau } \over \rm \tau }} \right)}^{ - \rm \tau }}} \right) \cr&\quad+ \mathop \sum \limits_{i:{y_i} \gt 0} \ln \left( {\rm \tau \left( {\displaystyle{{{{\it e}^{X_i^{\prime}{\rm \beta} }} + \rm \tau } \over \rm \tau }} \right) + {y_i}\ln \left( {1 + {{\it e}^{X_i^{\prime}\rm \beta }}\tau } \right)} \right) \cr&\quad+ \mathop \sum \limits_{i:{y_i} \gt 0} \ln \left( {{\Gamma }\left( \rm \tau \right)} \right) + \ln \left( {{\Gamma }\left( {1 + {y_i}} \right)} \right) - \ln \left( {{\Gamma }\left( {\rm \tau + {y_i}} \right)} \right),}$$

and the estimates of ZINB regression model are obtained by solving the log-likelihood using the iterative BFGS method (for more details on iterative BFGS method, see Fletcher (2013))

#### The multinomial logistic model

To see the effect of potential factors affecting the happiness of women after COVID-19, we have used multinomial logistic regression as the dependent variable was categorical. We also tested the assumption of parallel lines which confirmed the use of said technique and justified its preference over ordinal regression.

$$\eqalign{{\rm \theta} \left( {Y = k{\rm |}X = {x_{mi}}} \right) =&\,\,logit {\rm \delta} \left( x \right) = \ln \left[ {\displaystyle{{{\rm \delta} \left( x \right)} \over {1 - {\rm \delta} \left( x \right)}}} \right] = {{\rm \beta} _{ok}} + {{\rm \beta} _{1k}}{x_{1i}} \cr&+ {{\rm \beta} _{2k}}{x_{2i}} + {{\rm \beta} _{3k}}{x_{3i}} + \ldots + {{\rm \beta} _{nk}}{x_{ni}}.}$$

where “*Y*” is a vector for dependant variable having *k* outcomes and “*X*” is a vector for independent variables. The number of observations is given by “*i*” and “*m*” denotes the number of independent variables.

## Results

In our sample, on an average basis, women are married for around 14 years. The average age of our respondents (women) is 38 years ranging between 20 and 72 years. As far as the number of children is concerned, 47% of women in our data have two children. It is interesting to note that in our survey, the majority of the women are highly educated, as we document on an average basis women have 14 years of schooling and their husbands have 15 years of schooling. Perhaps the logical reasoning behind this number is an online survey; nonetheless, it provides an interesting insight into the mentioned phenomena. As mentioned earlier we are particularly interested to capture the dynamics for such a woman who is tech aware and can fill the online survey. Although, the generalizability of our research remains intact for those who cannot use technology or perhaps more domesticated women even if there is a limit to generalizability we are more interested here to identify the issue of violence and happiness for the particular groups of women who are able to use the technology to fill up the survey. The mean education of women’s fathers’ education is higher secondary school certification and for women’s mothers’ education is graduation. The years of schooling shows the level of education attained. In Pakistan, 10 years of schooling is equivalent to secondary school certification, 12 years of schooling is equivalent to higher secondary school certification, 16 years of schooling is equivalent to graduation, 18 years of schooling is equivalent to post-graduation and 22 years of schooling is equivalent to a doctorate. For the respondent’s earnings, we observe that around 27% of the women in the sample earn more than PKR 25,000. For living arrangements, we observe that around 25% of women live in an extended family, and 12% of women live in multiple family settings. To observe the geographic variation, we see 17% of women belong to rural areas. Table 1 presents a detailed summary statistic of the few demographic variables. Additionally, we observe from the data based on literature that there is significant rise in the number of women who reported such a violence (including physical, emotional and verbal) during lockdown. After the lockdown was announced, the incidence of women violence has increased exponentially (United Nations Women, 2020; Warraich, 2020). Therefore, we have a strong case here to observe what has caused the violence during the COVID-19 induced lockdown.

**Table 1:** Descriptive statistics (Source: calculations of the authors).

Variables	Range	Mean	Std. deviation	Frequency distribution
Woman age	20–72	37.54	10.589	
Husband’s age	22–79	41.86	11.443	
Woman’s years of schooling	0–22	14.21	4.018	
Husband’s years of schooling	0–20	9.33	5.129	
Father’s years of schooling	0–22	11.97	5.187	
Mother’s years of schooling	0–22	15.04	4.001	
Years of marriage	1–46	13.94	10.698	
Number of children	0–8	2.21	1.555	
Area				Urban (325) Rural (64)
Employment status				Full time working (332) Part time working (16) Unemployed (10) Not working (31)
Family setting				Extended family (98) Multiple family (45) Nuclear family (246)
Relationship with husband				Very poor (29) Poor (23) Neutral (48) Good (99) Very good (190)
Income adequacy				Strongly disagree (28) Disagree (64) Neutral (69) Agree (155) Strongly agree (73)
Feel empowered before COVID-19				Not very empowered (19) Not empowered (25) Slightly not empowered (36) Neutral (103) Slightly empowered (85) Empowered (72) Very empowered (49)
Say in social distancing decision				Strongly disagree (36) Disagree (22) Neutral (93) Agree (125) Strongly agree (113)
Feel anxiety				Strongly disagree (28) Disagree (84) Neutral (72) Agree (137) Strongly agree (68)
Feel empowered when husband is around				Strongly disagree (47) Disagree (47) Neutral (81) Agree (115) Strongly agree (99)
Childcare				Husband alone (10) Woman herself (63) Others (44) Together (woman and her husband) (72)
Access to police				Strongly disagree (93) Disagree (55) Neutral (108) Agree (68) Strongly agree (65)
Violence				Yes (252) No (137)
Psychological violence				Yes (133) No (256)
Access to personal protection equipment				Yes (287) No (102)
Medical insurance				Yes (131) No (258)
Decision making				Yes (302) No (87)
Happiness				Not very happy (42) Not happy (38) Slightly not happy (40) Neutral (57) Slightly happy (91) Happy (69) Very happy (52)
Number of observations	389			

### Violence on women during covid-19 related lockdown

In our sample, our calculations revealed that 65% of women (*N* = 257) have reported that they have not faced any violence during this lockdown, but the rest of the 35% (*N* = 132) revealed to suffer from some form of violence during the lockdown, and this primarily includes physical, emotional and verbal violence. In particular to physical violence, 83% of women no violence at all, but the remaining 17% reported having faced emotional violence up to 12 times during this lockdown. It is noteworthy that it has been 3 months since the Government has imposed the lockdown. The number of times women faced verbal and emotional abuse is higher than physical violence. Around 28% of women faced verbal violence, and 34% of women faced emotional violence. Verbal and psychological violence both contribute towards emotional violence where the women are not physically beaten but yet bruised mentally. According to WHO, emotional abuse is also part of intimate violence which is hurtful and usually not focused. Violent men use a range of strategies to exercise their power and control, which also includes emotional violence (García-Moreno et al., 2005). When a man cannot demonstrate himself as a successful man as expected by society, then he suffers from an identity crisis and uses violence to solve that crisis (Peterman et al., 2020). It is important to see the factors which are increasing the violence on women during the lockdown.

In this study, the number of violence is defined as the number of times a woman faced violence (i.e., physical/emotional/verbal). We applied Poisson, Negative Binomial, ZIP, and ZINB distributions to access the best-fitted count model for the number of violence. By following the goodness of fit criterions such as log-likelihood, Akaike Information Criterion (AIC), and Bayesian Information Criterion (BIC), which are reported in Table 2, we found that the number of violence is best fitted using the ZINB distribution as it showed minimum values of log-likelihood, AIC and BIC as compared to other models.

**Table 2:** Assessment of the best-fitted model for the number of violence.

Parameters	Poisson	Negative binomial	ZIP	ZINB
Location	3.90 (0.10)	3.90 (0.56)	2.41 (0.03)	2.13 (0.15)
Dispersion	–	0.13 (0.01)	–	0.49 (0.27)
Zero-inflation	–	–	0.61 (0.11)	0.15 (0.22)
Log likelihood	−2,872.50	−712.15	−1,539.66	−707.08
AIC	5,747.01	1,428.30	3,083.31	1,420.16
BIC	5,750.97	1,436.22	3,091.24	1,432.05

Further, to access the influential factors that affect the number of violence, the ZINB regression was applied, and the findings of the ZINB model are reported in Table 3. We found that age significantly affects the violence count, but the odds of elderly women having more frequency of violence are very less. This could be argued as the longevity of relationships perhaps increases the understanding and reduced the odds of having violence which even stays significant during the lockdown situation. The family setting in which a woman lives also plays an important role in violence where a woman can face more burden of responsibilities, strict enforcement of her assumed roles decided by the patriarchal system, and stereotyping. As compared to an extended family where a woman lives, the odds of having a higher frequency of violence are lesser in a nuclear family and multiple families. Around 25% of women in the sample live in an extended family setting and are more vulnerable to violence during this COVID-19 related lockdown. Around 42% of women reported taking care of children alone, and 55% of women perform household chores on their own.

**Table 3:** The zero-inflated Negative Binomial regression model for the number of violence.

Variables	Count model				Zero model			
	Estimate	Standard error	*z*-value	Odds	Estimate	Standard error	*z*-value	Odds
Intercept	1.50*	0.73	2.04	4.48	−10.50***	2.81	−3.74	0.00
Area (Base category: Rural)								
Urban	0.30	0.17	1.76	1.35	1.43**	0.64	2.23	4.17
Age	0.03**	0.01	2.29	1.03	0.11***	0.03	3.42	1.11
Years of education	−0.03	0.02	−1.34	0.97	0.18**	0.08	2.30	1.20
Family setting (Base category: Extended family)								
Multiple family	−0.93***	0.26	−3.63	0.39	0.48	0.83	0.58	1.62
Nuclear family	−0.78***	0.19	−4.02	0.46	−0.91	0.50	−1.83	0.40
Husband’s year of education	−0.01	0.02	−0.43	0.99	0.00	0.07	−1.83	1.00
Employment status (Base category: Working)								
Not Working	0.47***	0.18	2.54	1.60	1.99***	0.69	2.87	7.30
Part time	0.10	0.37	0.26	1.10	1.29	1.13	1.14	3.64
Unemployed	0.84***	0.30	2.78	2.31	1.86**	0.84	2.21	6.42
Number of children	−0.14	0.08	−1.85	0.87	−0.28	0.19	−1.45	0.76
Relationship with husband (Base category: Very poor)								
Poor	0.42	0.22	1.88	1.52	−2.67	1.86	−1.43	0.07
Neutral	0.13	0.28	0.44	1.13	−0.29	1.37	−0.21	0.75
Good	0.03	0.32	0.09	1.03	1.14	1.34	0.85	3.12
Very good	-1.16***	0.33	−3.54	0.31	1.54	1.29	1.20	4.67
Income adequacy (Base category: Strongly agree)								
Strongly disagree	0.50	0.32	1.59	1.65	0.41	1.07	0.39	1.51
Disagree	−0.06	0.26	−0.24	0.94	−0.69	0.97	−0.70	0.50
Neutral	−0.04	0.25	−0.17	0.96	0.20	0.61	0.33	1.22
Agree	0.07	0.22	0.33	1.08	−0.29	0.52	0.58	0.75
Feel empowered before COVID-19 (Base category: Not very empowered)								
Not empowered	−0.23	0.37	−0.61	0.80	−0.52	1.29	−0.40	0.59
Slightly not empowered	−0.28	0.37	−0.76	0.75	−1.54	1.20	−1.29	0.21
Neutral	−0.33	0.38	−0.89	0.72	−1.07	1.01	−1.06	0.34
Slightly empowered	−0.08	0.41	−0.19	0.92	−1.12	0.99	−1.13	0.33
Empowered	0.28	0.42	0.66	1.32	−1.08	0.99	−1.09	0.34
Very empowered	1.17***	0.47	2.47	3.23	−0.91	1.04	−0.87	0.40
Say in social distancing decision (Base category: Strongly disagree)								
Disagree	−0.86***	0.24	−3.57	0.42	0.48	1.52	0.32	1.62
Neutral	−1.30***	0.27	−4.75	0.27	0.57	1.10	0.52	1.77
Agree	−1.85***	0.29	−6.48	0.16	1.49	1.10	1.35	4.43
Strongly agree	−0.31	0.32	−0.98	0.73	2.07*	1.07	1.93	7.90
Childcare (Base category: Husband alone)								
Woman herself	0.83**	0.37	2.23	2.30	1.29	1.54	0.84	3.62
Others	1.05***	0.40	2.64	2.86	1.95	1.60	1.22	7.06
Together (woman and her husband)	0.74	0.40	1.84	2.10	1.81	1.55	1.17	6.13
Anxiety (Base category: Agree)								
Strongly disagree	0.65	0.46	1.40	1.91	1.60**	0.78	2.06	4.96
Disagree	0.57**	0.25	2.29	1.77	1.01*	0.56	1.81	2.74
Neutral	0.13	0.26	0.48	1.13	0.44	0.54	0.81	1.55
Strongly agree	−0.20	0.18	−1.09	0.82	−1.18*	0.69	−1.71	0.31
Feel empowered when husband is around (Base category: Agree)								
Strongly disagree	1.12***	0.29	3.82	3.06	0.52	0.89	0.59	1.69
Disagree	0.86***	0.27	3.23	2.36	−0.22	0.67	−0.33	0.80
Neutral	0.48**	0.24	1.99	1.61	0.08	0.53	0.15	1.08
Strongly agree	−0.11	0.29	−0.38	0.90	−0.07	0.55	−0.13	0.93
Log(theta)	1.30***	0.22	5.90	3.66				

**Notes:**

* *p*-value < 0.1.

** *p*-value < 0.05.

*** *p*-value < 0.01.

Odds = exp (estimate).

For zero violence, urban areas as compared to rural areas have more odds of having definite zero violence. Women having higher education have more odds of zero violence. Unemployed women and women who are not working have higher odds of zero violence as compared to the working woman. These women also have higher odds of having a higher frequency of violence.

As compared to women who are working, unemployed women and not working women have higher odds of having violence. It demonstrates that financial independence coming in complement with working plays an important role in avoiding violence. Additionally, we found the role of relationship women shares with her spouse plays a pivotal role in determining the violence. For instance, those women who have reported a very good relationship with their husbands as compared to very poor have lower odds of having violence during the lockdown. Working women during the lockdown faced a lot of trouble to fit in stereotypical roles, perform equally well in the job and managing housekeeping everyone safe. The struggle was hard for working women as compared to women who are not working or unemployed because they had the double burden of workplace and house management without house help. Her employment status may become a factor for her higher odds of violence. If everything is fine, unemployed and women who are not working will be not facing any violence, but they may also have higher odds of having a higher frequency of violence as compared to working women if things at her home or with her husband are not smooth.

This implies the role of anxiety in determining the violence, and this has been ascertained with the use of ZINB regression along with the count. Anxiety is also an important factor to affect violence. An anxious woman may involve in fight rejecting the patriarchal roles or asking the husband for helping her in responsibilities. She might not appear as a perfect and happy servant for the house all the time because of her own disturbed mental health and anxiety due to COVID-19. Women who do not have anxiety have higher odds of having zero violence. As compared to agreeing, if she disagrees with having anxiety, then still the odds of having violence are positive.

Empowerment of women is also important to reduce violence against them (World Health Organization, 2005). Women who were very empowered before COVID-19 as compared to those who were not very empowered have higher odds of having violence. The results seem surprising, but in the Pakistani context, they are not. Since very empowered women who were working (most probably) and taking decisions independently before the COVID-19 lockdown is now facing difficulty in accepting the stereotyping and taking care of children alone (after school closures) and without house help. This suggests that she is struggling to involve her husband in every responsibility and to shun the patriarchal norms. In this struggle, she has higher odds of facing violence. Women empowerment is often complimented with her role in household decision making. Therefore, we analyzed that as compared to those women who strongly disagree that they have “any say in social distancing”, women with more “say” have lower odds of having violence. As their autonomy in social distancing decision increase, the odds of having violence decreases. Childcare is a very important responsibility during this lockdown, especially when the orders for daycare closures and school closures are already in place. If a woman is performing this duty alone or others (for example relatives, woman’s parents, husband’s parents are doing it), then odds for a woman facing violence are higher as in both cases the husband’s involvement is zero which may become the cause of dispute. At the same time, if a woman is agreeing on feeling empowered when her husband is around, her odds of having violence is lower.

We investigated that the region, age, education, employment status, and anxiety are significantly affecting and predicting zero violence. In count model, we found that age, type of family setting, type of relationship with husband, feeling empowered before COVID-19, childcare responsibility, anxiety, and feeling empowered when the husband is around are important factors that affect the frequency of violence that women face. Living urban/rural regions, education of woman/husband, number of children, and adequacy of household income during this lockdown does not affect the count of women’s violence.

### The overall happiness of women after covid-19 related lockdown

The overall happiness of women after COVID-19 related lockdown is found to be affected by many factors. The assumption of parallel lines was checked to fit ordinal models, but a violation of it suggested the use of multinomial logistic regression. The results are detailed in Table 4.

**Table 4:** The multinomial logistic regression model for the happiness.

Variables	Models					
	(2)	(3)	(4)	(5)	(6)	(7)
Area (Base category: Rural)						
Urban	4.32** (1.15–16.21)	1.74 (0.46–6.56)	5.05** (1.03–24.87)	3.56* (0.94–13.47)	2.41 (0.59–9.76)	2.44 (0.53–11.19)
Age	0.98 (0.92–1.04)	1.01 (0.96–1.07)	0.99 (0.94–1.05)	0.98 (0.93–1.04)	1.00 (0.94–1.05)	0.94** (0.89–1.00)
Years of schooling	1.15 (0.96–1.38)	1.01 (0.85–1.21)	1.19* (0.99–1.43)	1.16* (0.98–1.37)	1.22** (1.02–1.47)	1.12 (0.92–1.37)
Family setting (Base category: Extended family)						
Multiple family	1.26 (0.20–7.84)	0.41 (0.03–5.45)	2.34 (0.35–15.74)	3.34 (0.53–21.11)	1.01 (0.12–8.50)	3.47 (0.44–27.05)
Nuclear family	1.60 (0.42–6.04)	1.98 (0.55–7.13)	1.73 (0.47–6.40)	3.14* (0.92–10.74)	2.52 (0.71–8.93)	3.46* (0.87–13.68)
Husband’s years of schooling	0.88 (0.75–1.04)	1.12 (0.93–1.36)	0.93 (0.78–1.12)	1.02 (0.87–1.20)	1.09 (0.91–1.30)	1.07 (0.88–1.30)
Income adequacy (Base category: Agree)						
Strongly disagree	0.17* (0.02–1.36)	0.33 (0.02–4.53)	0.50 (0.03–8.53)	0.56 (0.06–5.16)	0.21 (0.01–3.25)	2.46 (0.24–24.69)
Disagree	0.44 (0.11–1.76)	0.24* (0.05–1.23)	1.02 (0.23–4.53)	0.41 (0.10–1.71)	0.22* (0.04–1.09)	0.65 (0.10–4.06)
Neutral	0.23 (0.04–1.46)	1.10 (0.25–4.84)	1.27 (0.29–5.67)	0.40 (0.09–1.71)	0.13** (0.03–0.63)	0.72 (0.14–3.71)
Strongly agree	1.48 (0.17–12.65)	5.60 (0.70–44.58)	4.15 (0.50–34.60)	6.84* (0.92–51.15)	1.75 (0.22–14.13)	12.31** (1.46–103.70)
Access to police (Base category: Strongly disagree)						
Disagree	3.31 (0.64–17.19)	0.66 (0.10–4.26)	3.49 (0.54–22.70)	1.44 (0.25–8.28)	0.50 (0.07–3.66)	0.73 (0.08–6.35)
Neutral	0.94 (0.19–4.75)	0.60 (0.12–2.85)	2.28 (0.47–11.17)	0.93 (0.21–4.02)	0.96 (0.21–4.45)	0.74 (0.14–3.96)
Agree	2.84 (0.36–22.15)	3.23 (0.45–23.01)	8.06** (1.03–62.83)	3.17 (0.46–22.00)	3.70 (0.50–27.34)	4.67 (0.58–37.84)
Strongly agree	0.56 (0.09–3.59)	0.19 (0.03–1.44)	0.56 (0.08–3.82)	0.42 (0.08–2.19)	0.38 (0.07–2.17)	0.35 (0.06–2.11)
Anxiety (Base category: Agree)						
Strongly disagree	1.09 (0.04–31.35)	1.47 (0.05–43.72)	2.34 (0.10–54.97)	4.56 (0.26–80.85)	14.64* (0.82–261.60)	16.30* (0.91–291.60)
Disagree	0.37 (0.03–3.95)	0.58 (0.07–4.97)	3.53 (0.53–23.63)	4.83* (0.79–29.48)	9.81** (1.54–62.56)	8.50** (1.23–58.61)
Neutral	0.18 (0.01–2.76)	0.83 (0.12–5.92)	2.45 (0.37–16.18)	2.56 (0.40–16.25)	4.74 (0.72–31.41)	3.74 (0.51–27.60)
Strongly agree	0.14*** (0.04–0.52)	0.28* (0.07–1.12)	0.07*** (0.01–0.35)	0.12*** (0.03–0.48)	0.09*** (0.02–0.51)	0.12** (0.02–0.73)
Feel empowered when husband is around (Base category: Agree)						
Strongly disagree	1.51 (0.22–10.19)	0.35 (0.05–2.51)	0.00 (0.00–0.00)	0.06*** (0.01–0.45)	0.12** (0.02–0.91)	0.06** (0.01–0.56)
Disagree	3.47 (0.50–24.01)	2.11 (0.30–14.78)	1.56 (0.24–10.14)	0.65 (0.10–4.23)	0.97 (0.14–6.75)	0.60 (0.07–5.58)
Neutral	4.65 (0.70–30.88)	2.75 (0.46–16.35)	2.07 (0.37–11.47)	0.91 (0.17–4.87)	0.81 (0.14–4.73)	0.54 (0.08–3.74)
Strongly agree	2.04 (0.27–15.11)	1.24 (0.20–7.86)	0.54 (0.09–3.26)	0.92 (0.17–4.90)	1.05 (0.19–5.93)	1.25 (0.21–7.43)
Psychological violence (Base category: No)						
Yes	1.11 (0.29–4.29)	1.40 (0.38–5.18)	0.67 (0.18–2.51)	0.44 (0.13–1.55)	0.48 (0.13–1.83)	0.10*** (0.02–0.57)
Access to personal protection equipment (Base category: No)						
Yes	0.92 (0.24–3.59)	1.90 (0.39–9.40)	2.67 (0.39–18.15)	0.53 (0.13–2.21)	0.62 (0.12–3.10)	0.45 (0.08–2.50)
Medical insurance (Base category: No)						
Yes	0.29* (0.08–1.01)	0.38 (0.11–1.28)	0.48 (0.15–1.56)	0.42 (0.14–1.30)	0.39 (0.12–1.26)	0.55 (0.16–1.87)
Decision making (Base category: No)						
Yes	1.48 (0.67–3.27)	0.95 (0.42–2.14)	2.79** (1.26–6.17)	2.05* (1.00–4.20)	2.53** (1.19–5.38)	2.90*** (1.33–6.35)
Constant	0.49 (0.01–44.03)	0.09 (0.00–6.88)	0.00** (0.00–0.39)	0.09 (0.00–4.67)	0.01** (0.00–0.50)	0.05 (0.00–5.50)
Loglikelihood	−540.31***					
Pseudo R-square	0.27					
Test of parallel lines (loglikelihhod)^1^	100.90***					
Observations	389	389	389	389	389	389

**Notes:**

* *p*-value < 0.1.

** *p*-value < 0.05.

*** *p*-value < 0.01.

1 We tried to fit ordinal logit but the assumption of parallel lines was violated which suggested the use of multinomial logistic regression.

Relative risk ratio is shown for each variable along with standard errors in brackets.

The multinomial logistic regression shows model for not happy (2), slightly not happy (3), neither happy nor unhappy (4), slightly happy (5), happy (6), very happy (7). Not very happy (1) is the base outcome.

RRR, Relative risk ratio; SE, Standard error.

Using this technique, we found that women living in urban areas have higher odds of being happy than a woman living in a rural area during the lockdown period. Women locked down at home in urban areas have more access to utilities, facilities and have formal jobs which may make them happier. Age is significant to affect the happiness of women, but the odds are close to 1. Women having higher education have more odds of being happier than not very happy. Women living in nuclear family settings have higher odds of being overall happier than being not very happy. If the women strongly agree that household income is adequate for household expenditures, the odds of her being happy are higher. This implies the role of economic factors in determining happiness during the COVID-19 induced lockdown. If a woman agrees to have anxiety, then she has lower odds of being happier. Similarly, if she has emotional violence, then the odds of her being very happy are less. Since the happiness and psychological/emotional health are closely interrelated, thus, this finding further ascertains this phenomenon. If the woman’s role in decision making is more, then her odds of being happier are high. If the woman disagrees with feeling empowered when her husband is around, then her odds of being happier are significantly less. If a woman disagrees to feel empowered, then her odds of being happier are less.

During this lockdown after the COVID-19 pandemic, women living in urban areas, having higher education, having adequate household income to meet the expenditures, having lesser anxiety, not facing violence, feeling empowered when their husband is around, and have higher decision-making power are happier.

## Discussion and conclusion

Following WHO concerns of increased vulnerability of women amid the pandemic and calling for explaining the situation of women in specific contexts, we used the survey to investigate the situation of married women in Pakistan. Women staying at home are not safe, rather they are facing violence. They have higher odds of facing violence if they are locked down in an extended family setting, having higher number of children and having bad relationship with husband. The odds are also higher for unemployed women and those who are not working. If they are having anxiety, taking care of children alone and do not feel empowered if their husband is around then the odds of facing violence for women are higher. She is more enrooted in her patriarchal role assumed by society, and she has been endorsed firmly than ever. She is overburdened, anxious, and facing violence. In the 21st century and digital age, married women living in Pakistan are still not safe at homes where they are isolating themselves to avoid the disease.

Yet, the prevalence of violence does not mean that every woman is facing physical violence, so we also asked the woman about their subjective overall happiness after COVID-19. Our calculations ascertained that slightly over half of the women in our sample (around 53%) rated themselves as happy. One can possibly argue that the happiness of women while staying at home and being able to spend more time with family should have been increased but we observe around 47% of women reported otherwise. Their happiness after COVID-19 was found to be affected by the emotional violence that they face during COVID-19 related lockdown. Their anxiety, lesser decision making, lesser education, and living in a rural area are making them less happy during COVID-19 related lockdown. As it is evident from the debate and our findings that the role of gender is extremely important even during the COVID-19 related lockdown. Therefore, policy-making institutions should consider the mediating role of gender in relevant policymaking. A house is a women domain in the context of Pakistan, where she is mainly responsible for taking care of children and doing household chores. The Government should make policies reaching out to the women lockdown in their house, ensuring their health, safety, and psychological wellbeing. Empowering women can increase the effectiveness of the policies and women can play an important role during these times. Empowered women affect the lives of their families and children as they have more autonomy in decision making (Ewerling et al., 2017).

The study is important in the context of happiness and violence inflicted on women during the lockdown and provides the basis to improve the pandemic response policy. The authors do acknowledge the intrinsic limitations of the current study at this point. First of all, due to the lockdown situation, we had to rely on the online surveying approach, which might have some issues (i.e., inability to reach challenging population such as population living in remote areas and the majority of those women without technology awareness). Yet, this study revealed the vulnerability of women during lockdown staying at home. The irony is women are staying at home to be safe, but they are not safe at their homes from violence. This study conducted in Pakistan provokes the need for similar investigations in other countries where the stereotypical role of women is enforced, and gender equity is not ensured.

Factually, violence has affected the significant proportion of the population during the COVID-19 related lockdown. It threatens the lives and physical and mental health of people, overburdens health systems, undermines human capital formation, and slows economic and social development. Thus, one can argue that violence is predictable and therefore preventable. There are many other reasons which are extremely important to prevent the domestic violence for example no domestic violence may increase the subjective happiness and in turn higher productivity of women at work. Hence, preventing domestic violence is not only important from human rights perspective but it can also enhance the economic development and mental health of population. Prevention policies can be achieved through effective policing, strict legislation against violence, and through education.

## Supplemental Information

10.7717/peerj.10472/supp-1 Supplemental Information 1 Raw data.

10.7717/peerj.10472/supp-2 Supplemental Information 2 Questionnaire.

## Additional Information and Declarations

### Data Availability

The following information was supplied regarding data availability: The raw measurements are available in the Supplemental Files.
