# Supplementary material for: The pandemic paradox: domestic violence and happiness of women
Source: PeerJ. 2020 Nov 24;8:e10472. doi: 10.7717/peerj.10472 (PMC7694561; doi:10.7717/peerj.10472)
Supplement: Supplemental Information 2 [file peerj-08-10472-s002.pdf]

WOMEN DEALING WITH COVID-19 *All changes saved in Drive*

W

Questions Responses 412

WOMEN DEALING WITH COVID-19

This questionnaire is made to collect the responses of married women about how they are dealing with COVID-19 pandemic. Anonymity of respondent is assured.

Marital status *

- Currently married
- Divorced
- Widowed
- Other...

Who is the head of the household? *

- You
- Your husband
- Husband's Father
- Others

In which area do you live? *

- Urban
- Rural

*

Short answer text

Age (years) *

Short answer text

Husband's age *

Short answer text

Your years of schooling *

Short answer text

Which of the following best describes your family settings? *

- Nuclear family (husband, wife and children)
- Extended family (Husband, wife, children, grand children, their parents and any unmarried siblings)
- Multiple family (Husband, wife, children, grand children, their parents, parents in-law, married sibling and their child...

Husband's years of schooling *

Short answer text

Mother's years of schooling *

Father's years of schooling *

Short answer text

Your employment status *

- Full time
- Part time
- Unemployed
- Not working

Husband's employment status *

- Full time
- Part time
- Unemployed
- Not working

Spouse's Occupation *

Short answer text

Woman's occupation *

Short answer text

Short answer text

How many children do you have? *

Short answer text

Do you live in own house or live in rented one? *

- ○ Own a house
- ○ Rented house
- ○ Others

How would you rate your relationship with your husband? *

1 (Very Poor), 2 (Poor), 3 (Normal), 4 (Good), 5 (Very Good)

	1	2	3	4	5	
Very poor	○	○	○	○	○	Very good

Do you have any financial responsibility such as sharing the financial burden of the spouse? *

- ○ Yes
- ○ No

If you have the financial responsibility then how much do you contribute in family budget? *

- ○ Less than 10,000

- 20,001-30,000
- 30,001-40,000
- More than 40,000
- Not applicable

To what extent do you agree that woman should share the financial responsibility with their husband? *

- Strongly disagree
- Disagree
- Neutral
- Agree
- Strongly agree

What is your salary if you are working? *

Indicate 0 if you are not working

Short answer text

To what extent do you agree that your total income/pocket money is enough for you to meet your monthly living expenses during this COVID-19 related lockdown? *

- Strongly disagree
- Disagree
- Neutral
- Agree

To what extent do you agree that your household income (family income) is sufficient during this COVID-19 related lock down? *

- Strongly disagree
- Disagree
- Neutral
- Agree
- Strongly agree

To what extent do you agree that you are involved in economic decision making of the house during COVID-19 related lock down? *

- Strongly disagree
- Disagree
- Neutral
- Agree
- Strongly agree

Who decides for spending (household purchases) the money of the house? *

- Myself
- Husband
- Father
- Mother
- Others

To what extent do you think that you were empowered before COVID=19 related lock down? *

	1	2	3	4	5	6	7	
Not very empowered	○	○	○	○	○	○	○	Very empowered

To what extent do you agree that you have more say in implementing the social distancing? *

	1	2	3	4	5	
Strongly disagree	○	○	○	○	○	Strongly agree

To what extent do you agree that you have say in decision of health spendings in the house? *

- ○ Strongly disagree
- ○ Disagree
- ○ Neutral
- ○ Agree
- ○ Strongly agree

What do you think that which form of violence is justified? *

- ☐ Physical
- ☐ Verbal
- ☐ Psychological/emotional
- ☐ All of the above
- ☐ None

Did you ever fight with your husband in this lock down due to money? *

- Never
- Rarely
- Sometimes
- Often

How many times did you experience physical violence (hitting, beating, punching) during this lock down due to the current pandemic? *

Short answer text

How many times did you experience verbal violence (abusing) during this lock down due to the current pandemic? *

Short answer text

How many times did you experience emotional/psychological violence (ridiculing, bashing, not behaving/talking properly) during this lock down due to the current pandemic? *

Short answer text

In context of this pandemic, to what extent do you agree that you can approach police easily for help in case you experience violence? *

	1	2	3	4	5	
Strongly disagree	○	○	○	○	○	Strongly agree

- I am mainly responsible
- Husband
- Together (me and my husband)
- Others

Who is mainly responsible to do household chores during this lockdown? *

- I am mainly responsible
- Husband
- Together (me and my husband)
- Other...

Do you think that there are ways to engage both parents in childcare, domestic work or activities to support children's learning as schools are closed? *

- Strongly disgree
- Disagree
- Neutral
- Agree
- Strongly agree

Do you have sleep difficulty during this lock down? *

- Strongly disgree
- Disagree

- Agree
- Strongly agree

Do you think that you have anxiety during this lock down? *

- Strongly disgree
- Disagree
- Neutral
- Agree
- Strongly agree

Do you have equal access to face masks present in home during this lock down? *

- Yes
- No

Do you have equal access to gloves present in home during this lock down? *

- Yes
- No

Do you have equal access to hand sanitizer present in home during this lock down? *

- Yes
- No

Do you think that you can easily access to lady health worker or maternity hospital in this pandemic? *

- Strongly disgree
- Disgree
- Neutral
- Agree
- Strongly agree

Do you have any medical insurance provided by your employer or employer of your husband that you can use? *

- Yes
- No
- Maybe

Does both of you (you and your husband) inform each other about the pandemic and take decision together related to COVID-19? *

- Yes
- No
- I take alone as I am single
- Others

Were you tested for COVID-19? *

- Yes
- No

Do you think that your husband treated you/ will treat you badly when you exhibited/will exhibit symptoms of COVID-19 ? *

- Strongly disgree
- Disagree
- Neutral
- Agree
- Strongly agree

Do you think that you feel empowered when your husband (brother/father in case of being single) is around? *

- Strongly disgree
- Disagree
- Neutral
- Agree
- Strongly agree

Overall, how happy are you with your life nowadays after pandemic? *

	1	2	3	4	5	6	7	
Not very happy	○	○	○	○	○	○	○	Very happy

Overall, how happy you were before COVID-19 pandemic?

Linear scale

1 Not very happy

7 Very happy

Required
